# Evaluation of Stationary Wavelet Transforms in Reconstruction of Pure High Frequency Oscillations (HFOs)

**DOI:** 10.1007/978-3-030-51517-1_32

**Published:** 2020-05-31

**Authors:** Thouraya Guesmi, Abir Hadriche, Nawel Jmail, Chokri Ben Amar

**Affiliations:** 8 Digital Research Centre of Sfax, Sfax, Tunisia; 9 Institut Mines-Télécom, CNRS, Paris, France; 10 Université de Sherbrooke, Sherbrooke, QC Canada; 11 Digital Research Centre of Sfax, Sfax, Tunisia; 12 University of Sfax, Sfax, Tunisia; 13 Institut Supérieur d’Informatique et de Multimédia, Gabes University, Teboulbou, Tunisia; 14 Research Groups on Intelligent Machines Laboratory, REGIM, ENIS, Sfax University, Sfax, Tunisia; 15 Digital Research Centre of Sfax, Sfax University, Sfax, Tunisia; 16 Multimedia Information Systems and Advanced Computing Laboratory, MIRACL, Sfax University, Sfax, Tunisia

**Keywords:** IEEG signal, Epilepsy, HFOs, SWT, GOF, SNR

## Abstract

High frequency oscillations (HFO) from, MEG (magnetoencephalography) and intracerebral EEG are considered as effective tools to identify cognitive status and several cortical disorders especially in epilepsy diagnosis.

The aim of our study is to evaluate stationary wavelet transform (SWT) technique performance in efficient reconstruction of pure epileptic high frequency oscillations, reputed as biomarkers of epileptogenic zones: generators of inter ictal epileptic discharges, and offhand seizures.

We applied SWT on simulated and real database to detect non-contaminated HFO by spiky element. For simulated data, we computed the GOF of reconstruction that reaches for all studied constraint (relative amplitude, frequency, SNR and overlap) a promising results. For real data we used time frequency domain to evaluate SWT robustness of HFO reconstruction. We proved that SWT is an efficient filtering technique for separation HFO from spiky events. Our results would have an important impact on the definition of epileptogenic zones.

## Introduction

Over the past 15 years, researchers have shown usefulness of intra-cerebral activity above 70 Hz: High frequency oscillations (HFO) as efficient biomarkers for epileptogenicity. HFO has been considered as a clue of seizure build up and it appears especially, during ictal period. In fact HFO are exhibited as two sub band: ripples (80200) and fast ripples beyond 200 Hz [1].

HFOs can be detected visually by neurologist expert [2] or automatically: using automatic detectors developed on a preprocessing chain [3, 4].

To ensure the detection of HFOs, it is necessary to filter the used signal in HFO bands, however spiky component can disturb the detection stage due to induced false oscillations obtained by filter response [5]. Hence, it is necessary to choose accurate filtering technique within this framework.

Bénar et al. proved the efficiency of stationary wavelet transform in detection and separation between spikes and gamma oscillations.

Hence, we propose to study stationary wavelet transform performance in reconstruction of pure HFOs.

First, we tested SWT performance on filtered simulated data (in HFO frequency range) where we evaluated it for different constraints (SNR, overlap rate, relative amplitude and frequency range).

Second, our focus was to evaluate SWT robustness of HFO reconstruction on filtered real IEEG signal (in HFO frequency range) using time frequency analysis. These results would assist neurologist during diagnosis of pharmaco resistant patient by defining epileptogenic tissue that should be delineated through a surgical intervention.

In the first section, we depict our simulated and real data, the filtering technique and evaluation methods used. In the second section, we exhibit our obtained results and finally we conclude and discuss our results.

## Materials and Methods

### Materials

All signal-processing steps of our paper are executed using Matlab software (Mathworks, Natick, MA) with EEGLAB toolbox.

#### Simulated Data:

Obtained by a combination of a spike, and HFO shapes as real IEEG signal, sampled at 1000 Hz. Through different tests, we created different sets of signal (composed of spikes and HFO) by varying different parameters: relative amplitudes, frequency of oscillations, signal to noise ratio (SNR) and overlapping rate: we obtained 4 sets of simulated data composed of spiky and HFO events. We increased the spiky amplitude by 2, 4, 6, 8 and 10 times compared to oscillatory one. We varied also oscillation’s frequency in this range [80 150 100 200 250] Hz (ripples and fast ripples). Overlap between spike and HFO oscillations is changed with equal steps via the size of oscillations window: no overlap (spike and oscillation are completely separated) until we reached 100% overlap when spiky and oscillatory events are superimposed. The overlap step is equal to 25%. Finally, we ranged SNR ratio (Eq. **1**) from −5 dB to 20 dB.

1 $$ {\text{SNR}} = 10*{ \log }\left( {{\text{S}}/{\text{N}}} \right) $$

Where S is the simulated signal and N is the studied added noise.

#### Real Data:

(IEEG) recordings for a pharmaco-resistant epileptic subject, where acquisition and pretreatment steps were assigned to clinical neurophysiology department of La Timone Hospital, Marseille [6] and validated by an expert neurologist. Our data is recorded on a Deltamed system, sampled at 1000 Hz with a low-pass filter. This particular IEEG signal is selected since it exhibits important epileptic HFOs patterns and regular spikes.

### Methods

The Stationary Wavelet Transform SWT technique is a diversity of Dynamic Wavelet Transform with an advantage of overcoming decimation of DWT; which leads to a better maintain of signal characteristics. It performs even better than Continuous Wavelet Transform CWT by exceeding frequency-overlapping band.

In fact, SWT was used in various fields of application such as de-noising and detection [8], also, very useful in physiological signal analysis [7].

In [9] SWT was studied and implemented to reconstruct pre-ictal gamma oscillations in order to predict seizure build up. Hence, we proposed to study and evaluate SWT method performance in reconstruction of ripples and fast ripples: HFO. SWT decomposed a signal to be filtered into approximations and details coefficients, then, and through a thresholding steps (using masks), it allows to detect only desired parts by inverse of SWT method (iswt) [10]. Our thresholding step consists of creating a rectangle mask with a width equal to raw window studied and a length equal to 2 scales of decomposition (approximation and detail coefficients) [6]. SWT is a projection of scale $\theta_{j,k}$ function dilated and translated to obtain $cA_{j} (k)$ as approximations coefficients and $cD_{j} (k)$ as detail ones.

During implementation steps of SWT, we choose 6 levels of decomposition for a better detection of HFO [6]. We adopted the symlet wavelet family, since they are almost symmetrical to oscillation; moreover they are featured by their orthogonally which facilitate the reconstruction step.

#### Evaluation by Goodness of Fit

(GOF) is used in different areas to evaluate performance of filtering technique [6, 11, 12]. After applying SWT we computed GOF between reconstructed simulated signals (reconstructed HFO) s_r_(t) and original simulated signals (original simulated HFO) s(t) by the following formula:

2 $$ {\text{GOF}} = 1- \, \left( {\left( {{\text{sum}}\left( {{\text{s}}\left( {\text{t}} \right) - {\text{s}}_{\text{r}} \left( {\text{t}} \right)} \right)^{ 2} } \right)/{\text{sum}}\left( {{\text{s}}\left( {\text{t}} \right)} \right)^{ 2} } \right) $$

To evaluate SWT filtering method robustness in recovering pure HFO, we calculated similarity rate of reconstructed HFO within original simulated HFO signals for different frequency range, relative amplitude, overlap rate and (SNR).

#### Time Frequency Representation:

Obtained by a time-frequency transform that provides 2 dimensional domain of an original one dimensional signal. This card allows via visible inspection or thresholding step to define specific shape both in time and frequency plan in our case, we will define pure HFO from spiky events [13].

## Results

### Simulated Data

**Fig. 1.**
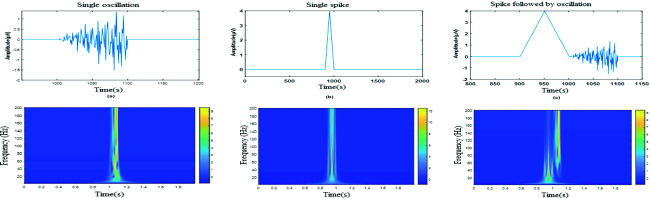
Time representation of (a) single oscillation, (b) single spike, (c) spike followed by HFO, proceeded successively by their Time frequency plan.

We depict in Fig. 1 three types of simulated data, where upper line is a temporal representation of a ripple, a spike and a spiky event with a ripple one. The lower line represent frequency plan of the studied events. Combining HFO and spiky event produces a complex shape in which it is difficult to distinguish basic elements.

**Fig. 2.**
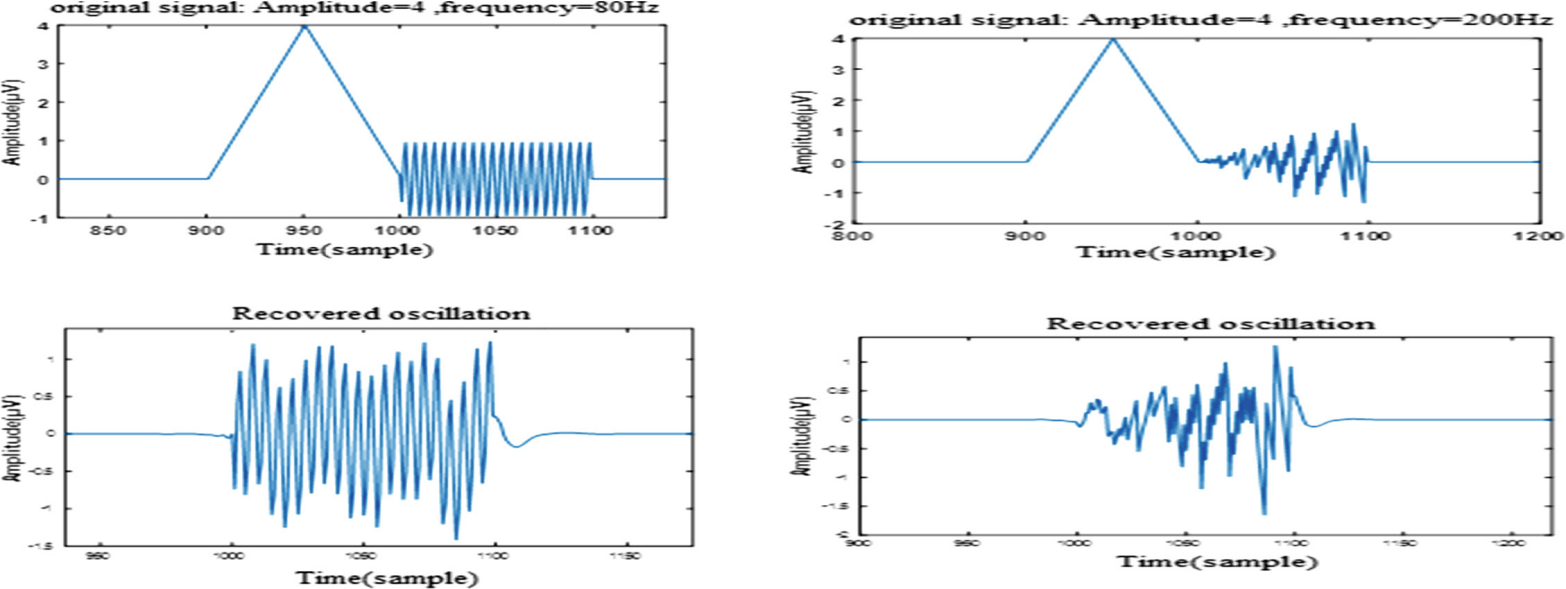
Two sets of reconstruction of HFO (80 Hz and 200 Hz) by SWT, first line is original signal, second line is recovered HFO.

In Fig. 2, we illustrated reconstruction of HFO by SWT technique for two frequency configurations; we studied different frequency effect on HFO reconstruction. SWT was able to reconstruct HFO with a minimum of spiky elements.

**Fig. 3.**
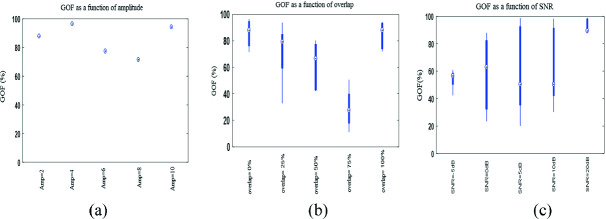
GOF values for (a) different relative amplitudes of spiky and HFO events, (b) different temporal overlap and (c) different SNR rates.

In Fig. 3(a), we gathered GOF values for HFO reconstruction after varying different relative amplitude between spiky and HFO events. We notice that for all relative amplitude, reconstruction result of HFO is beyond 78% for an amplitude of spiky event 8 times higher than HFO, and it reaches 97% for a relative amplitude equal to 4.

Hence, we can annotate that SWT have a good performance in reconstruction of HFO even for a low relative amplitude between spiky and HFO events.

We represent in Fig. 3(b) result of SWT reconstruction GOF for time overlaps constraint between spiky and HFO. We found that all configurations of overlap the GOF is beyond 80% for a low rate of overlap and total overlap, however the GOF rate declines for a rate of overlap >50% and <75%. We presented GOF as a box, where central mark is median, and 25th and 75th percentiles are edges of each box.

In Fig. 3(b), we displayed GOF result of HFO reconstruction as a function of SNR.

The best rate of GOF are obtained for SNR > 10 DB which exceeds 85%, however GOF results are lower for low SNR with a median GOF around 58% and 75th percentiles above 82%.

### Real Data

**Fig. 4.**
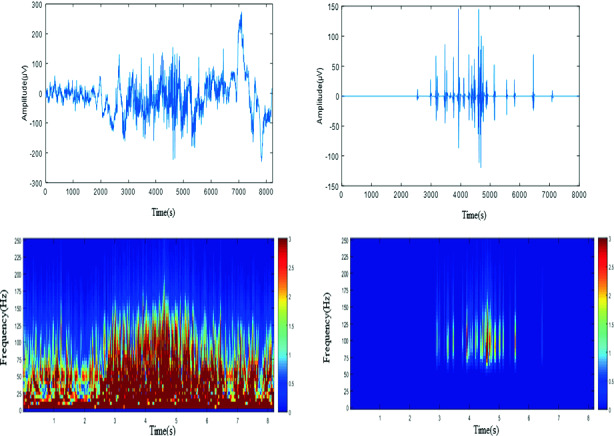
Upper line: real data (channel 26), and pure recovered HFO by SWT, lower line time-frequency representation of raw, and pure HFO.

In Fig. 4, we depict time series of our real data (channel 26) and its representation in time frequency domain (upper line). Our choice (channel 26) was justified by high occurrence of HFO comparing to other channels (important activities: spiky events and different rhythms of oscillations and HFO in full overlap). We filtered our real data in HFO frequency range than we applied SWT, and we depict pure recovered. In lower line we illustrate raw signal and recovered HFO time frequency domain.

After applying SWT, only ripples and fast ripples (HFO) are sustained, oscillatory activities with a frequency range beyond 80 Hz, which is clear from time frequency illustration. Hence, HFOs are efficiently reconstructed, by visual inspection of time frequency domain. SWT is a powerful method for separating such a range of oscillations frequency from spikes.

## Conclusion

In our study, we tested performance of SWT filtering method, in reconstruction of pure HFO (HFO without spiky events) on simulated data and real signal. For simulated signal (inspired from IEEG recording), we evaluated robustness of SWT in HFOs reconstruction for several parameters that are proved very powerful in varying quality of obtained results. We studied effect of relative amplitude between HFO and spiky events, impact of frequency range (ripples and fast one), overlapping rate and finally signal-to-noise ratio. From our obtained results and for all studied constraint, SWT reconstruction of HFO reveal a good GOF rate. Hence, such study could be as a reference of SWT performance in reconstruction of HFO among 4 constraints (amplitude, frequency, SNR, overlap).

For real data, we evaluated performance of SWT reconstruction of HFO using time frequency representation and neurologist expert inspection.

SWT gives good results in reconstruction of non-contaminated HFO by spiky element even in bad environment (low SNR, different range of frequency, high overlap and low relative amplitude). These results predispose SWT for reconstruction and detection of all range of oscillatory frequency. Our obtained results are very promising in further study of HFO and its networks connectivity since we are dealing with pure HFO and hence non-contaminated cortical generators. Our perspectives are defining pure HFO networks connectivity in order to define epileptogenic zones. This result would have an important impact on surgical intervention of pharmaco- resistant patient to delineate epileptogenic tissue and get free seizure.
